# Randomized comparative study of child and caregiver responses to three software functions added to the Japanese version of the electronic Pediatric Quality of Life Inventory (ePedsQL) questionnaire

**DOI:** 10.1186/s41687-020-00213-w

**Published:** 2020-06-23

**Authors:** Iori Sato, Mariko Sakka, Takafumi Soejima, Sachiko Kita, Kiyoko Kamibeppu

**Affiliations:** 1 Department of Family Nursing, Division of Health Sciences and Nursing, Graduate School of Medicine, the University of Tokyo, Tokyo, Japan; 2 Department of Health Quality and Outcome Research, Division of Nursing System, Global Nursing Research Center, Graduate School of Medicine, the University of Tokyo, Hongo 7-3-1, Bunkyo-ku, Tokyo, 113-0033 Japan; 3 Department of Gerontological Home Care and Long-term Care Nursing, Division of Health Sciences and Nursing, Graduate School of Medicine, the University of Tokyo, Hongo 7-3-1, Bunkyo-ku, Tokyo, 113-0033 Japan

**Keywords:** Children, Family, Patient-reported outcomes, Parent, Pediatrics, Practical report, Quality of life, Randomized controlled trial

## Abstract

**Background:**

Patient-reported outcomes (PROs) refer to any report of the status of a patient’s health condition, health behavior, or experience with healthcare directly from the patient, without interpretation of the patient’s response by a clinician or any other external party. While many PROs, such as the Pediatric Quality of Life Inventory (PedsQL), were originally administered in paper-and-pencil format, these are now available as electronic versions (ePROs). Although ePROs might well have used the same structure as their paper versions, we developed an alternate ePedsQL incorporating three software functions: 1) a non-forcing non-response alert, 2) a conditional question branch of the School Functioning Scale that only displays for (pre) school children, and 3) a vertical item-by-item display for small-screen devices. This report evaluated the effect of these functions on item non-response rate, survey completion time, and user experience.

**Methods:**

All surveys were conducted via the online/computer mode. We compared the dynamic format containing the three functions with the basic format in a randomized comparative study in 2803 children and 6289 caregivers in Japan.

**Results:**

We found that the non-response alert lowered the item non-response rate (0.338% to 0.046%, t = − 4.411, *p* < 0.001 by generalized linear mixed model analysis). The conditional question branch had mixed effects on survey completion time depending on the respondents’ age. Surprisingly, respondents rated the vertical question display for handheld devices less legible than the matrix format. Further, multigroup structural equation modelling revealed that the same configuration for both formats showed an acceptable fit (CFI 0.933, RMSEA 0.060, SRMR 0.038) but the errors of observed variables were larger for the dynamic format than the basic format.

**Conclusions:**

We confirmed the robustness of the ePedsQL in different formats. The non-response rate of ePedsQL was very low even in the absence of an alert. The branch and item-by-item display were effective but unnecessary for all populations. Our findings further understanding of how humans respond to special software functions and different digital survey formats and provide new insight on how the three tested functions might be most successfully implemented.

## Background

Patient-reported outcomes (PROs) refer to any report of the status of a patient’s health condition, health behavior, or experience with healthcare directly from the patient, without interpretation of the patient’s response by a clinician or any other external party [1, 2]. These are valuable tools in clinical and research settings to gauge patients’ perceptions and feelings [3–7]. In pediatrics, regardless of the cognitive limitations of infants, children, and adolescents, outcome evaluation by children themselves (child self-report) is recommended whenever possible [8–10]. Parent-proxy report is recommended together with child self-report [8–10] from a person- and family-centered care standpoint, as is patient and family engagement in healthcare [2]. Assessment and feedback using PRO surveys can improve patient/guardian communication with physicians and reduce the number of unidentified problems in clinical settings for children with cancer [11–13] and juvenile idiopathic arthritis [14]. In research settings, PROs are the most desirable method for evaluating subjectively-defined symptoms, such as fatigue, nausea and severity of pain [1, 15]. PROs (including proxy-reported outcomes) are widely embedded in research and clinical settings [16, 17].

Most PROs were developed as paper-and-pencil questionnaires, but electronic versions of PROs (ePROs) can improve their usability [13, 18–21]. Paper-and-pencil questionnaires have logistical costs, including printing and the time/labor required to review responses, that ePROs can minimize. Furthermore, electronic questionnaire systems have special capabilities beyond what written tests can achieve. It is important to note that the U.S. Food and Drug Administration (FDA) advises clinicians to test the response equivalence of new ePROs to paper-and-pencil questionnaires, especially if the structure or format of an ePRO differs from the original paper version [14, 22]. Although software-specific functions may improve the user experience and outcomes of ePROs, researchers have not evaluated if and how reporters respond differently with and without these platform-specific functions.

One of the most frequently used PRO measures for children is the Pediatric Quality of Life Inventory (PedsQL) [23–25]. The PedsQL requires participants to answer items about children’s health-related quality of life. The survey varies in length and rating scale based on the child’s age. Although the PedsQL was originally developed and validated by paper-and-pencil [15, 26, 27], an electronic version (ePedsQL) has been released and its equivalence has been confirmed in various settings [28–30]. Although this ePedsQL is already available [31], it uses the same format as the original paper version. We wanted to determine whether adding special software functions could further improve survey outcomes. To investigate this question, we incorporated three software functions into our ePRO system. We then assessed the similarities and differences in how reporters—both children and parents—reacted to an ePedsQL with and without these dynamic functions to determine whether these functions should be introduced to ePROs in clinical and research settings.

The three functions we incorporated into the ePedsQL survey were 1) a non-forcing non-response alert, 2) a conditional question branch of the School Functioning Scale that displays only for (pre) school children, and 3) a vertical item-by-item format display, rather than a matrix format, for small-screen devices (details of these functions are provided in the Methods section). The first special function was a non-forcing non-response alert. When patients and caregivers answer PRO questions, they sometimes leave questions unanswered, either accidentally or intentionally. We hypothesized that adding a non-forcing non-response alert would lower the accidental item non-response rate but would still allow users to intentionally skip questions if they chose to.

The second software function added to our dynamic ePedsQL survey was a conditional branch of questions. In the PedsQL there is a 3-item section called the School Functioning Scale. Children aged 6 years old or younger and their caregivers only answer this section if the child goes to (pre)school; if these criteria do not apply, they continue on without responding to this section. The dynamic ePedsQL survey asked reporters for the school status of children aged 6 years or younger and only displayed the School Functioning Scale if the child attends (pre)school. Therefore, because not all questions are shown to all reporters, we expected that implementing a conditional question branch would decrease the required survey completion time.

The third and unique and intentional software function was a vertical item-by-item format display. Because participants completed the ePedsQL evaluations on their own personal devices, which included personal computers, tablets, and smart phones, this allowed us to investigate whether reporters respond differently to alternate survey formats on different types of devices. This type of data is valuable in a practical sense because the ePedsQL may in fact be administered on different devices. The basic ePedsQL (presented in the same structure as the paper version of PedsQL) and the dynamic ePedsQL for wide-screen devices use matrix placement of questions and response options in the same horizontal row. We hypothesized that switching to an item-by-item format (with questions and response options listed vertically) on small-screen devices would improve the subjective legibility and make it easier for survey participants to select a response. We therefore tested the measurement invariance when the survey was given in the item-by-item format, relative to the standard ePedsQL matrix format.

There is accumulated evidence from previous studies on the effect of such format/functional changes on people’s reporting [32–36]. However, the level of evidence is mixed. and the International Society for Pharmacoeconomics and Outcomes Research identified different levels of equivalence evaluation using the following methods in order of evidence and burden: cognitive debriefing<usability testing<equivalence testing<full psychometric testing [22]. Advancements in programming technology have recently led to the use of many types of functions becoming common practice, even without evaluation. Particularly in children, because of their lower accessibility and vulnerability, evaluation studies are rare and have lower levels of evidence. We expect that our study will be important for accumulating strong evidence (usability testing and full psychometric testing) for ePRO research in children and their caregivers.

In this study, we identify the similarities and differences between child and caregiver responses and between narrow and wide-screen devices in response to the basic and dynamic versions of the ePedsQL. The basic version of the ePedsQL was presented and functioned in the same way as the paper version of the PedsQL (i.e. responders can move forward without providing a response). Meanwhile, the dynamic version of the ePedsQL possessed three dynamic characteristics of online/computer surveys. We evaluated survey outcomes by measuring non-response rates, survey completion times, and subjective legibility. Here we report our findings and make recommendations on what software functions should be incorporated under which circumstances to improve electronic PRO surveys.

## Methods

We conducted a randomized controlled trial comparing the responses by both children and their caregivers to two different formats of the ePedsQL (with and without dynamic functions). The study protocol was reviewed and approved by the Ethics Committee of the Graduate School of Medicine, University of Tokyo. This study was registered to the UMIN Clinical Trial Registry (UMIN000031311).

### Participants

We recruited children and their caregivers for the study from two different sources in February and March of 2018. Children who were 1 month to 18 years old were the target participants of this study; however, children aged 5 to 18 years were invited because of the age range of the PedsQL. Caregivers were invited if they had children aged between 1 month and 18 years. Two family caregivers for each child were included in the study as the “primary caregiver” and “secondary caregiver” because most children in Japan have two caregivers in their families [37]. Primary and secondary caregivers were defined as family caregivers by candidate participants (parents) who were invited to participate this study. The candidate participants indicated whether their relationship to the child was primary or secondary caregiver on the recruitment website.

The first recruitment site was an internet survey company. We chose a large-sized company with a balanced panel that continuously improved the panel by restricting incorrect/conflicting responses. Registrants of the company who reported having a child aged 1 month to 18 years at the time of registration were invited by e-mail to complete an online questionnaire for this study. If the respondent had two or more children, only one child was considered. Each user received only one invitation, which was non-replicable because of an attached identification number (ID). The company tried to continue the e-mail invitation until the sample size of each age (0-year, 1-year, 2-years, … 18-years) were achieved 100.

The second recruitment site was the authors’ neighborhoods. This site was selected to increase the sample size through recruitment of available participants. We recruited survey participants by snowball sampling in which we hand-delivered leaflets about the online questionnaire system to people interested in the study. If the participants had two or more children, the corresponding number of leaflets was delivered—one leaflet each with a unique ID number per child.

We recruited participants from these two groups to ensure that we had a sufficiently large sample size and a variety of characteristics among participants. We predicted that internet survey company users may be conditioned to online/computer surveys and neighborhood participants may have characteristics similar to those of the researchers. We initially treated these two groups of participants together as a complementary mixture, and subsequently conducted subgroup analysis to examine the consistency of results between the groups.

### Procedure

All surveys were conducted via the online/computer mode. Candidate participants from both recruitment sites were able to log into the online system on their own devices (personal computers, tablets, smartphones, etc). They were informed about this study on the website. If they gave consent, they entered their child’s birthday, sex, primary caregiver’s relationship to the child (mother, father, etc.) and secondary caregiver’s relationship (father, mother, nonexistent, etc). The “nonexistent” option was only allowed for secondary caregivers because there had to be a primary caregiver but not a secondary caregiver. The system checked that the child’s age was between 1 month and 18 years old.

The survey system randomized participants into two groups: one that received the basic survey format and one that received the dynamic version equipped with special software functions. Respondents were stratified based on the child’s age and participant recruitment site. The randomization ratio was 1:1 and the block size was two.

After randomization, participants (primary caregivers, secondary caregivers, and children) separately answered their own questionnaires. If there was no secondary caregiver, the corresponding questionnaire was not shown. If the child was younger than 5 years old, the child questionnaire was not shown.

The entire survey comprised two webpages: The first comprised the ePedsQL (explained in detail below) and the second comprised questions about user experience and sociodemographic characteristics. The candidate participants (from both the internet panel and neighborhoods) were paid recompense based on determination by the survey company that they had completed all of the survey pages.

### Basic ePedsQL survey format

The PedsQL survey varies in length and rating scale based on the child’s age as follows: The Generic Core Scales [26, 27] require 8–18-year-olds and their caregivers to evaluate 23 health-related items using the 5-point Likert scale. Caregivers of 2–7-year-olds respond to only 21 items. Children of 5–7 years old evaluate these 21 items based on a 3-point face scale, rather than the 5-point scale. The Infant Scales require caregivers of 1–12-month-olds and 13–24-month-olds to evaluate 36 and 45 items, respectively [38].

We used the Japanese versions of these scales which have been translated and validated and are widely-used for Japanese children and their caregivers [31, 39–41]. In Japan, education is compulsory for children over 6 years old, while children aged 6 years old or younger can choose whether to go to preschool (kindergarten, daycare, etc). The last 3 of the 21 items in the PedsQL Generic Core Scales for 2–7-year-olds and/or their caregivers comprise a School Functioning Scale, which must only be evaluated for children going to (pre)school. Therefore, a directive message is written before the last 3 items for children aged 6 years old or younger and their caregivers: “Please answer the next section only if you (your child) go to (pre)school”.

**Fig. 1:**
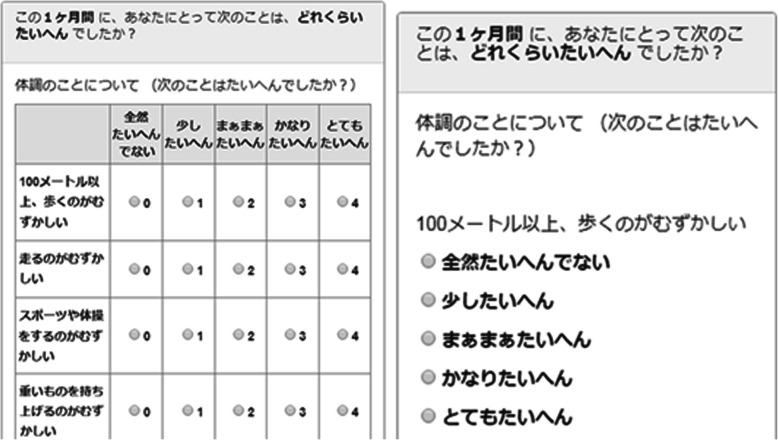
General appearance of the matrix survey format and the vertical item-by-item display for small-screen devices. Actual appearance varied by user device

We programmed the basic format of the ePedsQL to match the original PedsQL in structure, including using matrix placement (see Fig. 1) of questions and response options. All response options were placed in the same horizontal row as each question.

### Dynamic ePedsQL survey format

We added three special software functions into our ePedsQL questionnaire system and refer to it as the dynamic format of ePedsQL. The first special function was a non-forcing non-response alert. When patients and caregivers answer PRO questions, they sometimes leave questions unanswered. There are two categorical reasons for non-response: 1) questions are forgotten or overlooked, or 2) questions are unanswerable or the reporter hesitates to answer. We can expect that a non-response alert will decrease the former. However, a non-response alert that forces a reporter, who would otherwise leave a question unanswered for the latter reason, to provide an answer does not respect the person’s right to privacy and to choose not to answer a question. Therefore, a non-response alert was displayed in our questionnaire, but reporters could choose whether they went back to answer a question or continued forward with a non-response.

The second advanced software function added to our dynamic ePedsQL survey was a conditional branch of questions. In the PedsQL School Functioning Scale for children aged 6 years old or younger and their caregivers, reporters must read the directive message to answer only if the child goes to (pre)school; if these criteria do not apply, they continue on without responding to the 3 items. Therefore, our dynamic ePedsQL survey asked reporters for the school status of any child younger than 6 years before showing the School Functioning Scale, and then displayed the last 3 questions only if the child attended (pre)school.

The third advanced function to be added to the dynamic ePedsQL survey was an item-by-item format for small-screen displays (Fig. 1). Handheld devices have recently been developed and spread over the world. When an ePRO is in a grid format, the font size of the questions and the response option buttons become too small to read or select on small devices. We thought that an item-by-item display of questions and response options would be more legible and easier to answer on small screens. All response options were listed vertically beneath each question. Our dynamic ePedsQL obtains data on the width of the Internet browser being used at all times and automatically transforms the matrix format used for devices wider than 600 pixels (wide-screen devices) to the item-by-item format on devices with a pixel-width less than 600 (small-screen devices).

### Survey evaluation

One of the primary measures of efficacy for this study is the non-response rate of answerable items. The number of answerable items for 8–18-year-olds and their caregivers was 23; for 5–7-year-old (pre) school children and their caregivers was 21; for 2–5-year-olds not attending (pre) school and their caregivers was 18; for 13–24-month-old children’s caregivers was 45; and for 1–12-month-old children’s caregivers was 36. If the School Functioning Scale items were left unanswered in the basic ePedsQL survey for children aged 6 years old or younger, it was assumed that they did not attend (pre)school.

There were two additional evaluative outcomes. The first was the required time to complete the ePedsQL, which was measured from when participants opened the ePedsQL web-page (the full questionnaire was displayed on one page regardless of the number of items) to when they closed the ePedsQL web-page. Soon after they closed the ePedsQL web-page, the following (user experience and sociodemographic characteristics) web-page was automatically opened. The ePedsQL formats were also evaluated based on the user-response survey presented on the web-page following the questionnaire, where responders rated 6 questions about the survey’s subjective legibility on a 7-point scale: (i) Overall, how hard was it to answer the survey? Answer ‘1’ if not hard at all, ‘7’ if very hard; (ii) How visible were the characters? Answer ‘1’ if very easy to see, ‘7’ if very difficult to see; (iii) How appropriate was the size of characters? Answer ‘1’ if too small, ‘4’ if appropriate, ‘7’ if too big; (iv) Did you understand the meaning of questions easily? Answer ‘1’ if very easy to understand, ‘7’ if very difficult to understand; (v) How easy was it to select the response options? Answer ‘1’ if very easy, ‘7’ if very difficult; (vi) Are your eyes tired now? Answer ‘1’ if not tired at all, ‘7’ if very tired.

### Statistical analyses

All analyses were conducted using R 3.5.1 [42]. The *p*-value threshold of significance was set to 0.05. The second author (MS) masked participants’ survey groups by assigning them meaningless symbols and the first author (IS) analyzed the data in this blinded manner until results were finalized.

A primary analysis of non-response frequency was carried out using a generalized linear mixed model (GLMM). Each item that was answered by a responder was coded to ‘0’ and each non-responded item was coded to ‘1’. The observations were nested by each reporter, the reporters were nested by each child, and the children were nested by each family (4-level hierarchical model). Item-, reporter-, child-, and family-level variance were calculated and used to indicate the origin of non-response (i.e. item difficulty, reporter’s attentiveness, child’s lack of expression). The GLMM estimated the non-response rates of basic and dynamic ePedsQL formats, and tested whether or not the two rates differed.

There was an important consideration regarding the analysis of the survey-completion time: since participants answered the ePedsQL on their own devices, they were able to break in the middle of answering the survey. The apparent time required to complete the ePedsQL, as measured, could thus become as long as overnight or more. For this reason, restricted mean survival time (RMST) was calculated as if participants who took more than 20 min to complete the survey within the study period—a time determined by previous studies [39, 43–46]—had completed it in 20 min. Participants who did not complete their surveys by 23:59 on March 31, 2019 were considered censored subjects for the analysis.

We used the Mann-Whitney U test to compare the subjective legibility of the two survey formats. Subgroup analyses were conducted separately for children and caregivers with respect to the above analyses (non-response frequency, survey completion time and subjective legibility) as follows: survey company users and residents of researchers’ neighborhoods; 0–6 and 7–18-year-old children; device screen less than 600 pixels wide and those greater than 600 pixels wide.

Measurement invariance between the two survey formats was tested by multigroup structural equation modelling [47, 48]. A 5-factor structure of PedsQL Generic Core Scales reported by children was established and confirmed by previous studies [27, 39, 49–51]. This structure was assumed here and configurational invariance was checked by the goodness of model fit (Good fit: comparative fit index (CFI) > 0.95, root mean square error of approximation (RMSEA) < 0.05, standardized root mean square residual (SRMR) < 0.05; Acceptable fit: CFI > 0.9, RMSEA < 0.08, SRMR < 0.08) [52–54]. To test a measurement’s invariance, we applied the following equality constraints between two groups sequentially: (i) factor loadings, (ii) intercepts of observed variables, (iii) means of latent variables, (iv) errors of observed variables, (v) variances of latent variables, (vi) covariances of latent variables [47, 48]. Application of more equality constraints led to poorer model fit. When the decrease in model fit became too marked, the equality constraint was judged to be inapplicable. We therefore calculated the degree of CFI decrease (ΔCFI) by each equality constraint. We considered an equality constraint not applicable when ΔCFI > 0.02 [55]. If equality constraint (i) was applicable, metric invariance between the two survey formats was confirmed. Further, if equality constraint (ii) was applicable, scalar invariance was confirmed. Metric and scalar invariance between groups are necessary to determine that two survey formats are psychometrically equivalent. We checked constraints (iii) to (vi) with no hypothesis (exploratory analysis).

The minimum required sample size was determined from a 2 × 2 comparative Fisher’s exact test (based on non-response events and group allocation) because, to the best of our knowledge, there is no sample size calculation for GLMM analysis. This sample size test is used in the special case that an event rate is very low [56]. The calculated sample size of children was 1249 per group, for a power level of 0.8, two-sided α error level of 0.05, non-response rate to the basic ePedsQL format of 0.8% determined by a previous study [39], and non-response rate to the dynamic ePedsQL of 0.1%. The calculated sample size of caregivers was 1508, which was based on the same values, except that the non-response rate to the basic ePedsQL was assumed to be 0.7% for adults [39].

## Results

### Participants

**Fig. 2:**
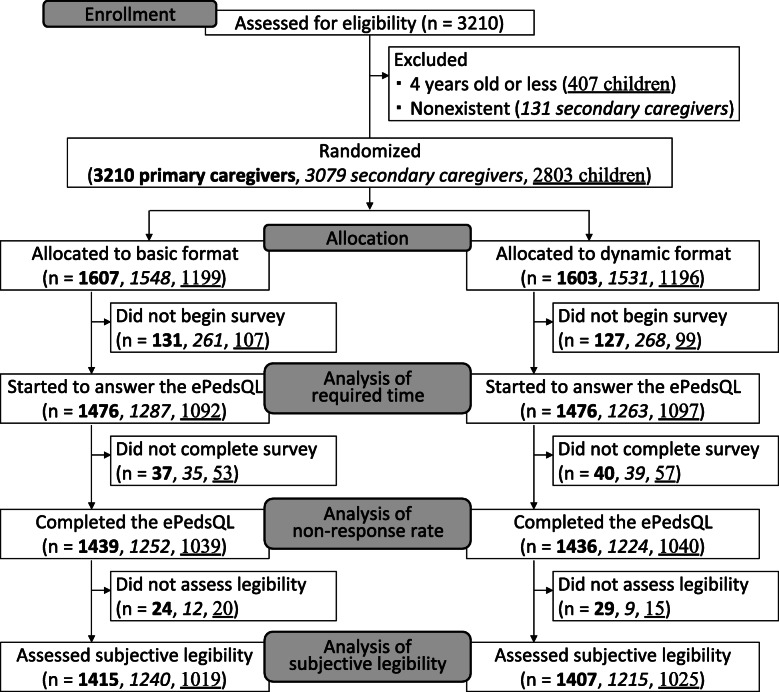
Flow of participants: Primary caregivers, secondary caregivers and children. Bold, italic, and underlined numbers are the number of primary caregivers, secondary caregivers, and children, respectively

For recruitment of participants through the internet survey company, the company distributed invitation emails to registered users regardless of their eligibility for this study. Of those invited, 2529 caregivers with a 1-month to 18-year old child consented to participate. To recruit participants by snowball sampling, the authors hand-delivered 1555 survey invitation pamphlets, first to their direct neighbors, then to interested individuals within their neighbors’ social networks. Of the hand-delivered pamphlets, 681 children’s caregivers consented to participate. Accordingly, a total of 3210 families were enrolled in this study. Of the 3210 families, all families had primary caregivers, whereas only 3079 families had secondary caregivers. Further, 407 of 3210 children were 4 years old or younger. Therefore, 3210 primary caregivers, 3079 secondary caregivers, and 2803 children were randomly allocated into the two survey groups (basic and dynamic ePedsQL formats) (Fig. 2).

Of the primary caregivers (1607 allocated to the basic format and 1603 allocated to the dynamic format), 2952 (1476 and 1476) primary caregivers started to answer the ePedsQL (group used to analyze the survey completion time), 2875 (1439 and 1436) completed the survey and continued on to the second web-page (group used to analyze the non-response rate), and 2822 (1414 and 1407) completed the second survey for evaluating the subjective legibility of their ePedsQL format (group used to analyze subjective legibility). Similarly, 2455 (1240 and 1215) secondary caregivers and 2044 (1019 and 1025) children completed the ePedsQL and legibility survey (Fig. 2).

Completion rate after randomization in the basic and dynamic format groups was 88% (1415/1607) and 88% (1407/1603) among primary caregivers, 80% (1240/1548) and 79% (1215/1531) among secondary caregivers, and 85% (1019/1199) and 86% (1025/1196) among children, respectively. Therefore, the probability of dropout was comparable between the randomized groups. Further, the two groups remained comparable after participant dropout in terms of recruitment source, children’s age, health status, caregiver’s relationship to child, education level, working status and display used, but not gender of the primary caregiver’s child (*P* = 0.034 by Fisher’s exact test) (Table 1). Participant’s characteristics are summarized by recruitment source in Supplementary Table 1.

**Table 1:** Participant characteristics

	Children				Primary caregivers				Secondary caregivers			
	Basic		Dynamic		Basic		Dynamic		Basic		Dynamic	
	*N* = 1019		*N* = 1025		*N* = 1415		*N* = 1407		*N* = 1240		*N* = 1215	
	n	(%)	n	(%)	n	(%)	n	(%)	n	(%)	n	(%)
Recruitment												
Survey company	836	(82)	844	(82)	1097	(78)	1093	(78)	1015	(82)	994	(82)
Researchers' neighborhoods	183	(18)	181	(18)	318	(22)	314	(22)	225	(18)	221	(18)
Children's age												
0-1 years old	-	-	-	-	123	(9)	126	(9)	94	(8)	97	(8)
2-4 years old	-	-	-	-	253	(18)	241	(17)	211	(17)	203	(17)
5-6 years old	164	(16)	157	(15)	174	(12)	176	(13)	151	(12)	146	(12)
7 years old	69	(7)	73	(7)	74	(5)	76	(5)	65	(5)	67	(6)
8-12 years old	369	(36)	378	(37)	375	(27)	373	(27)	337	(27)	241	(28)
13-15 years old	204	(20)	206	(20)	203	(14)	208	(15)	186	(15)	179	(15)
16-18 years old	213	(21)	211	(21)	213	(15)	207	(15)	196	(16)	182	(15)
Children's gender												
Female	526	(52)	480	(47)	697	(49)	658	(47)	613	(49)	564	(46)
Male	493	(48)	545	(53)	718	(51)	749	(53)	627	(51)	651	(54)
Children with any disease which requires clinic/hospital visit												
Yes	123	(12)	121	(12)	175	(12)	157	(11)	152	(12)	131	(11)
No	873	(86)	876	(85)	1232	(87)	1241	(88)	1080	(87)	1075	(88)
Unknown (not answered)	23	(2)	28	(3)	8	(1)	9	(1)	8	(1)	9	(1)
Caregiver relationship to child												
Mother	-	-	-	-	1370	(97)	1357	(96)	35	(3)	38	(3)
Father	-	-	-	-	35	(2)	42	(3)	1094	(88)	1063	(87)
Grandmother	-	-	-	-	10	(1)	8	(1)	91	(7)	98	(8)
Grandfather	-	-	-	-	0		0		11	(1)	6	(0)
Other	-	-	-	-	0		0		9	(1)	10	(1)
Caregiver’s education level												
Junior high school	-	-	-	-	18	(1)	14	(1)	33	(3)	40	(3)
Senior high school	-	-	-	-	287	(20)	317	(23)	259	(21)	297	(24)
Vocational college	-	-	-	-	189	(13)	193	(14)	155	(13)	135	(11)
Junior college	-	-	-	-	295	(21)	271	(19)	45	(4)	35	(3)
University	-	-	-	-	527	(37)	516	(37)	584	(47)	567	(47)
Graduate school	-	-	-	-	74	(5)	67	(5)	146	(12)	118	(10)
Unknown (not answered)	-	-	-	-	25	(2)-	29	(2)-	18	(1)	23	(2)
Caregiver’s working status												
Working	-	-	-	-	742	(52)	759	(54)	1141	(92)	1096	(90)
Not working	-	-	-	-	648	(46)	619	(44)	81	(7)	96	(8)
Unknown (not answered)	-	-	-	-	25	(2)	29	(2)	18	(1)	23	(2)
Display size												
Greater than 600 pixels	594	(58)	633	(62)	726	(51)	751	(53)	667	(54)	682	(56)
Less than 600 pixels	425	(42)	392	(38)	689	(49)	656	(47)	573	(46)	533	(44)

### Outcome analyses

The overall item non-response rate to the basic ePedsQL was 0.338% and that to the dynamic format was 0.046%. The percent of responders who had one or more non-response items was 3.7% and 0.3% for the basic and dynamic formats, respectively. GLMM analysis showed that the family-level variance was 2.5*10^− 6^, child-level variance was 2.4*10^− 16^, reporter-level variance was 8.1*10^− 3^ and item-level variance (residual) was 1.1*10^− 3^. The GLMM test also showed that the overall item non-response rates significantly differed (t = − 4.411, *p* < 0.001). Subgroup analysis showed that the non-response rate of the dynamic ePedsQL was lower than that of the basic format in all subgroups (Table 2).

**Table 2:** Item non-response rate of two formats of the ePedsQL

	Basic format		Dynamic format		Difference	Ratio
	n	%	n	%	%	
Children						
All children	1039	0.420	1040	0.011	0.41	38
Children of survey company users	856	0.500	859	0.010	0.49	50
Children from researchers’ neighborhoods	183	0.047	181	0.016	0.03	2.9
0–6-year-old children	164	0.203	157	0.000	0.20	∞
7–18-year-old children	875	0.461	883	0.013	0.45	35
Displays greater than 600 pixels	654	0.608	686	0.006	0.60	101
Displays less than 600 pixels	385	0.101	354	0.020	0.08	5.1
Caregivers						
All caregivers	2691	0.306	2660	0.060	0.25	5.1
Survey company users	2142	0.296	2120	0.025	0.27	12
Researchers’ neighbors	549	0.345	540	0.195	0.15	1.8
0–6-year-old children’s caregivers	1024	0.271	1004	0.029	0.24	9.3
7–18-year-old children’s caregivers	1667	0.328	1656	0.079	0.25	4.2
Displays greater than 600 pixels	1520	0.290	1551	0.026	0.26	11
Displays less than 600 pixels	1171	0.327	1109	0.107	0.22	3.1

**Fig. 3:**
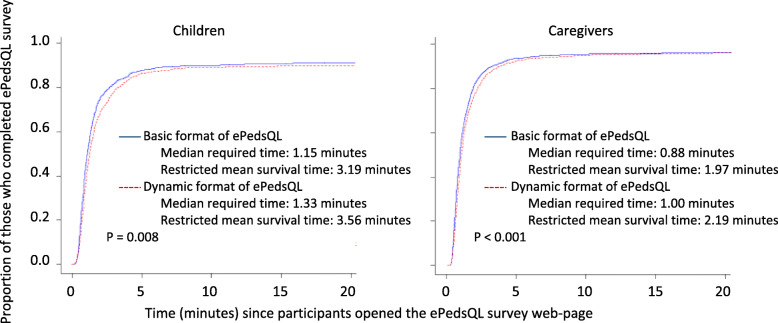
Kaplan-Meier curve showing survey response time for two different ePedsQL formats for children (left) and caregivers (right). The Kaplan-Meier curves show the cumulative proportion of reporters who completed the ePedsQL survey and the time to completion from the time they opened the web-page. If the curve is shifted toward the left and top, this indicates that more reporters completed the survey in a shorter period of time

The survey completion time for the ePedsQL was about 3 min for children and 2 min for caregivers. The dynamic format took longer to complete than the basic format, both for children and caregivers (Fig. 3) and in all subgroup variations (Table 3). However, 0–6-year-old children needed very little extra time to complete the dynamic survey format. Caregivers recruited from snowball sampling and caregivers who used a narrow-screened device required more time to complete the dynamic ePedsQL than participants recruited through the survey company and those who used a wide-screened device.

**Table 3:** Survey completion time (minutes) of two formats of the ePedsQL

	Basic format			Dynamic format			Difference		Ratio	
	n	Median	RMST	n	Median	RMST	Median	RMST	Median	RMST
Children										
Children of survey company users	902	1.03	2.77	908	1.17	3.12	0.1	0.4	1.1	1.1
Children from researchers’ neighborhoods	190	2.60	5.19	189	3.00	5.69	0.4	0.5	1.2	1.1
0–6-year-old children	168	1.36	3.70	165	1.40	3.89	0.0 ^a^	0.2	1.0 ^a^	1.1
7–18-year-old children	924	1.13	3.10	932	1.32	3.50	0.2	0.4	1.2	1.1
Displays greater than 600 pixels	610	0.95	1.76	646	1.06	2.23	0.1	0.5	1.1	1.3
Displays less than 600 pixels	429	1.42	3.15	394	1.72	3.36	0.3	0.2	1.2	1.1
Caregivers										
Survey company users	2170	0.78	1.69	2150	0.87	1.73	0.1	0.0 ^a^	1.1	1.0 ^a^
Researchers’ neighbors	556	1.68	3.05	549	2.12	3.98	0.4	0.9	1.3	1.3
0–6-year-old children’s caregivers	1038	1.22	2.45	1025	1.33	2.73	0.1	0.3	1.1	1.1
7–18-year-old children’s caregivers	1688	0.75	1.67	1674	0.85	1.85	0.1	0.2	1.1	1.1
Displays greater than 600 pixels	1417	0.73	1.42	1454	0.77	1.53	0.0 ^a^	0.1	1.1	1.1
Displays less than 600 pixels	1274	1.11	2.08	1206	1.35	2.40	0.2	0.3	1.2	1.2

“Difference” (median or restricted mean) refers to the time required for the dynamic format minus that for the basic format. For example, children of survey company users required a median of 1.17 min for the dynamic format and 1.03 min for the basic format. Therefore, the difference was calculated as 0.14 and rounded to 0.1. A difference greater than 0 indicates that the dynamic format requires extra time for completion

“Ratio” (median or restricted mean) refers to the time required for the dynamic format divided by that for the basic format. In the example using children of survey company users (median of 1.17 min for the dynamic format and 1.03 min for the basic format), the ratio was calculated as 1.136 and rounded to 1.1. A ratio greater than 1 indicates that the dynamic format requires extra time for completion

*RMST* Restricted Mean Survival Time

^a^Difference close to 0 or ratio close to 1 are notable results

**Fig. 4:**
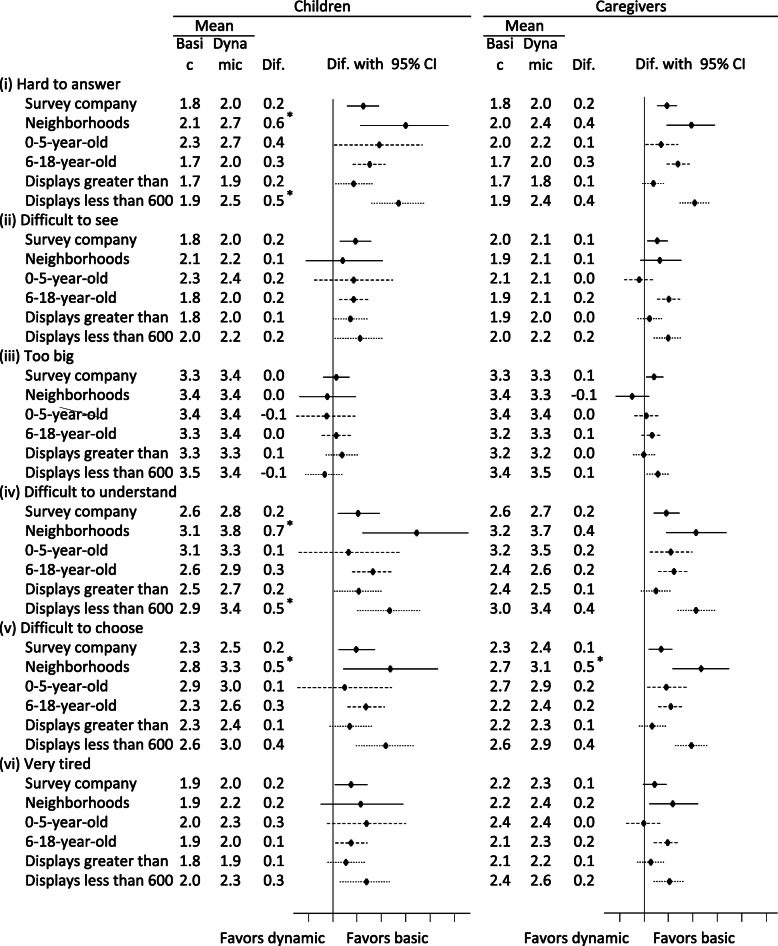
Subgroup analysis of the subjective legibility of two formats of ePedsQL questionnaires. Full question and response options: (i) Was it hard to answer, based on your overall impression? Answer 1 if not hard at all, 7 if very hard; (ii) How visible were the characters? 1 if very easy to see, 7 if very difficult to see; (iii) How appropriate was the size of characters? 1 if too small, 4 if appropriate, 7 if too big; (iv) Did you understand the meaning of questions easily? 1 if very easy to understand, 7 if very difficult to understand; (v) How easy was it to select the response options? 1 if very easy, 7 if very difficult; (vi) Are your eyes tired now? 1 if not tired at all, 7 if very tired. CI: confidence interval. Dif: Difference. If the difference between the mean value in the basic format and dynamic format was greater than 0, this indicates that the reporters favored the basic format over the dynamic format. For example, children of survey company users reported an average of 2.0 for illegibility for the dynamic format and 1.8 for illegibility for the basic format. Therefore they favored the basic format by 0.2 points. The 95% confidence intervals are also shown. Intervals further to the right mean the basic format was favored. * Difference in mean illegibility reported between basic and dynamic formats > 0.5 points

Most children and caregivers reported that both formats of the ePedsQL were legible (Supplementary Table 2). Except for the letter size, children reported that the dynamic format was less legible than the basic format. Caregivers also reported that the dynamic format was less legible, but by a smaller margin than the children. Additionally, in each subgroup, children and caregivers consistently tended to report that the dynamic format was hard to answer, difficult to see, difficult to understand, contained difficult-to-choose options, and caused their eyes to be very tired (Fig. 4). Particularly obvious differences (0.5 or greater) between the two formats were consistently observed in the neighborhood subsample and reporters using narrow devices. Between subgroups, children and caregivers who completed the surveys on narrow devices consistently considered the dynamic format (item-by-item display) to be less legible across almost all of the legibility questions.

### Measurement invariance

**Fig. 5:**
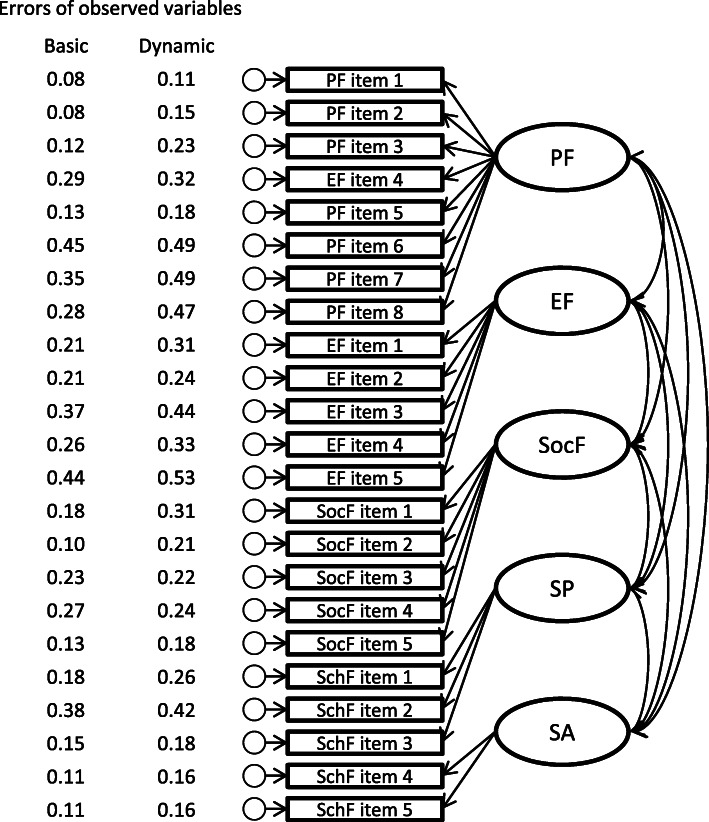
Estimates by multigroup structural equation modelling with equality constraint of all estimates except errors of observed variables. EF: Emotional Functioning. PF: Physical Functioning. SA: School Absenteeism. SchF: School Functioning. SocF: Social Functioning. SP: School Presenteeism. In one case, the multigroup structural equation model showed that the basic and dynamic ePedsQL had similar (equally constrained) structure (shown in this figure) and metrics (path coefficient, intercept, variance and covariance) but not errors (variances) of the observed variables (each item). Accordingly, each error of the observed variables was estimated as shown in the figure by group

**Fig. 6:**
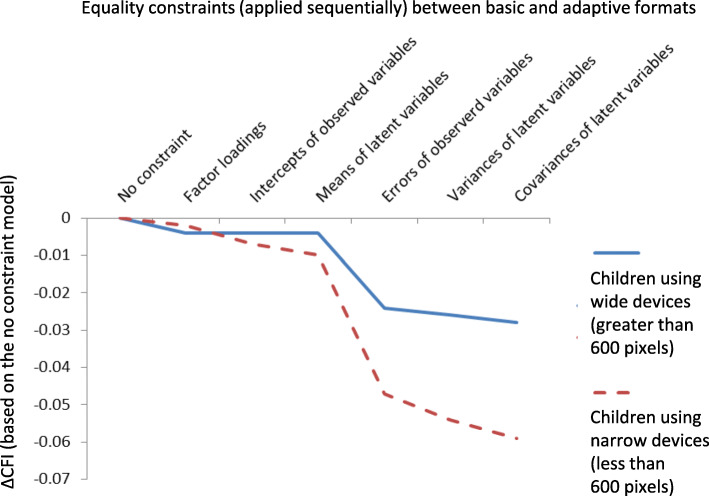
Effect of equality constraints on ΔCFI for small and large-screen devices. CFI: comparative fit index. Based on the first model (no constraints), each sequential constrained model showed that the goodness of model fit (CFI) decreased (ΔCFI). ΔCFI > 0.02 was used to indicate that the equality constraint is not applicable. Factor loading (path coefficient from latent variables to observed variables), intercepts of observed variables (estimated average for each item), and mean of latent variables (estimated average of measured concept (QOL subscales)) were judged to be comparable between basic and dynamic formats. Errors of observed variables (variance of each item) among children using narrow-screen devices (less than 600 pixels) were judged to be different to those among children using wide-screen devices (greater than 600 pixels)

Multigroup structural equation modelling under the same configuration for both formats showed an acceptable fit (CFI 0.933, RMSEA 0.060, SRMR 0.038). After applying equality constraints to the models including factor loadings, intercepts of observed variables, and means of latent variables, group outcomes were found to be equal (ΔCFI: 0.001 to 0.002). However, the ‘errors of observed variables’ equality constraint did not support equal group outcomes (ΔCFI = 0.021). Therefore, we constructed a model with all of the equality constraints except the ‘errors of observed variables’ (Fig. 5) and found that nearly all of the errors of observed variables were larger for the dynamic format than for the basic format. Post-hoc subgroup analyses showed that the measurement variance in the observed variables originated in small displays, which had an item-by-item format in the dynamic ePedsQL (ΔCFI = 0.036, Fig. 6).

## Discussion

The purpose of this study was to gain an understanding of how children and caregivers react to a dynamic version of the ePedsQL compared to a basic version that is equivalent to the original paper-and-pencil survey format. Survey participants who took the dynamic ePedsQL survey had a lower non-response rate, took more time to complete the survey, and reported that the dynamic version was comparatively less legible than the basic format. Response analyses confirmed the scalar and metric invariance in outcomes from the two survey formats. Additionally, we found that there were greater errors of the observed variables in the dynamic ePedsQL.

### Sample characteristics

We combined the data from the two recruitment groups because we predicted that they would have complementary characteristics. We predicted that internet survey company users may be conditioned to online/computer surveys. We found that (Supplementary Table 1) internet survey company users tended to answer the survey using wide-screen displays. Given the potential for similar trends in results from subgroup analysis by recruitment source and display size, results from subgroup analyses should be interpreted with caution. On the other hand, we predicted that participants from researchers’ neighborhoods may show characteristics similar to those of the researchers. Indeed, the neighborhood sample and researchers both had younger aged children, children with any disease, caregivers with high education level, and caregivers who were working. We performed subgroup analyses to examine the consistency of results between the two recruitment groups. Although there were some consistencies (lower non-response rate, time-consuming nature, illegibility, and metric invariance of the dynamic format), the levels varied. A discussion on this is provided below.

We were able to uniformly collect data on the children’s age and gender. According to the Japanese national survey, the proportion of children visiting a clinic/hospital is 15% [57]. Internet panel users tended to not have children with any disease, which complemented the finding in the neighborhood sample. Among families with children in the Japanese national survey, 87% of fathers and 67% of mothers are working [57]. There is a discrepancy in this parameter between our sample and the national survey. This may be because non-working caregivers tend to be primary caregivers to children and working caregivers tend to be secondary caregivers, which suggests that the proportion of primary caregivers who are working is lower than that of working mothers, and that the proportion of working secondary caregivers is higher than that of working fathers. In another census [58], 36% of men and 22% of women in their 30s had university-level education, while the proportion was lower among those in their 40s and 50s. The sample in this study clearly had higher education levels.

### Interpretation of results

Decreased non-response rate was an expected and natural result of the non-response alert introduced to the dynamic ePedsQL. According to the variance at each of the 4 hierarchical levels examined, non-response mainly originated from reporter-level factors. Further, the non-response rate after the non-response alert was not zero. Non-responses due to forgotten or overlooked questions decreased while non-responses due to unanswerable questions or responder hesitation remained. This means that the non-response alert, without forcing a responder to provide an answer, functioned in an ethically appropriate manner, as expected.

Throughout all tested groups, the dynamic ePedsQL took longer to complete than the basic format, which likely resulted from the added non-response alert. Notably, preschool-aged children did not take much longer to complete the dynamic survey than the basic format. The conditional branch of questions was expected to shorten response times for young children who did not attend school and their caregivers. The conditional question branch did appear to shorten the survey completion time for young children but not for their caregivers. It is not clear why the caregivers of preschool-aged children did not have shorter survey response times. The sudden appearance of a different type of question (about a child’s schooling status) may have slowed or confused caregivers. If caregivers affirmed that the child attends school, they might also have been momentarily surprised by seeing three new questions appear.

Another possible explanation for the increased response time to the dynamic ePedsQL survey may be the different item-by-item structure. Previous studies have shown that adults take more time to complete item-by-item surveys than matrix-formatted surveys [32, 33]. This is consistent with the result that caregivers using narrow-screened devices took more time to answer the dynamic survey than those using wide-screened devices. However, there was no clear difference in survey response time for children taking the dynamic survey on narrow-screened versus wide-screened devices. This is the first study to report children’s response times to ePROs in different formats on different screen types.

Contrary to our initial hypothesis, both children and caregivers rated the legibility of the dynamic ePedsQL lower than the basic format. Based on the subgroup analysis, this may have been due to the item-by-item format implemented on narrow devices, especially among the neighborhood subsample. The neighborhood subsample was predicted to be less familiar with online/computer surveys than the survey company subsample. Although the survey company subsample has likely experienced various types of survey questionnaires, the neighborhood subsample may have felt uncomfortable with repeating the same response option. Another possible explanation is the increased screen scrolling required in the item-by-item format compared to the matrix format. However, a previous study in adults with psychological illnesses found that they preferred the item-by-item survey format over the matrix format [32]. Although individual preferences and survey legibility may differ between the previous study and our present study, this discrepancy should be investigated in future research.

It may be important to note that this study was conducted in Japan and in the Japanese language. Japanese characters can be written vertically and horizontally, unlike other (i.e. alphabetic) languages, which are only written horizontally. One characteristic of the item-by-item structure in this study was a decrease in line breaks (Fig. 1); however, this characteristic is unlikely to be important to people who are accustomed to reading multi-directional text. Thus, the findings in this study may have been affected by the specific reading capabilities of Japanese people [59] and may not be generalizable to other (e.g. European) cultures. We suggest that further research using eye-tracking technology may be effective for determining the importance of such capabilities.

We confirmed the metric and scalar invariance for children’s responses to both survey formats. The comparable factor loadings indicate that the overall concept was similar between the two survey formats, and the comparable intercepts of observed variables indicate similar reported average values. Confirmation of the metric and scalar invariance suggests that the two ePedsQL formats can be treated as similar psychometric tests, despite the difference in format and function. Importantly, the ‘means of latent variables’ equality constraint was satisfied for the two formats. We expected equivalent responses, as observed, because the ePedsQL format assignments were randomized. There were larger errors associated with the observed variables in the dynamic format, which we attribute to the item-by-item format on small-screen displays. The metric and scalar invariance in item-by-item and matrix-formatted surveys has been previously reported for adults [32, 34, 35]. However, this study is the first to report on the scalar and metric invariance in children. Previous studies in adults showed that the errors of observed variables were larger for matrix than for item-by-item formats [34, 36], which is the opposite of what we found for children. This indicates that children may respond differently to ePROs than adults, and serves as preliminary knowledge about children’s reaction to ePROs.

### Implications

The three dynamic functions added to the ePedsQL survey did not improve the overall user-experience for reporters. To determine whether to introduce new functions to a clinical ePRO, we must consider the purpose and expected outcome of each function, based on the findings of the present study and previous studies.

In this study, the non-forcing non-response alert decreased the non-response rate, but the decrease may not be clinically meaningful. Surprisingly, the non-response rate in this study was very small for both formats; previous studies using the paper-and-pencil PedsQL reported that non-response rates were 0.7–1.6% in children and 0.7–1.0% in caregivers [39, 43, 52]. Considering that the non-response rates for all forms of the ePedsQL used in our study were below 0.7%, clinicians should consider adopting the electronic version of PRO surveys—even without a non-response alert—for both children and caregivers to reduce non-response rates. Our findings report data for mostly healthy children and caregivers; it remains to be determined whether the observed trends apply to other groups.

In instances where the non-response rate is expected to be high, a non-response alert is useful for improving response rates. It is important that the alert does not force users to provide answers. When clinicians/researchers develop and administer PRO surveys, they must respect the right of a reporter not to answer a question. The non-forcing alert is one way to achieve both goals: it reduces accidental non-response while allowing intentional non-response. Researchers can choose to add a non-response alert (general alert, non-forcing alert) or not to add an alert to an ePRO according to the research regulations and population of the study with reference to these results.

This study showed that introducing a conditional branch of questions can sometimes increase survey completion time, although the degree of increase was very small. The PedsQL School Functioning Scale only has 3 response items and whether they need to be answered depends only on one condition (school status). Such a simple circumstance may not benefit from a conditional branching function which may momentarily slow responders. To reduce potential reporter confusion, it may be better to implement conditional branching over sequential survey pages.

Contrary to our expectation, the item-by-item display for narrow devices resulted in poorer outcomes in children with respect to survey completion time, subjective legibility, and answer error. Our initial supposition that the matrix format would become too small to read and answer on a smartphone was proved false. Relatively healthy children and their caregivers might not feel the matrix format on a small device is illegible in the first place. Understanding responses to conditional branching of questions and item-by-item displays can reduce programming costs.

Because metric and scalar invariance between the dynamic and basic ePedsQL formats was achieved, our study supports knowledge that the ePedsQL has psychometric robustness and is not highly sensitive to format changes. Our study confirms that the ePedsQL is a useful ePRO for children.

### Limitations

We calculated RMST by preliminarily setting it at 20 min prior to conducting the analysis. However, some reporters (even those that reported completing the survey within 20 min) completed the questionnaire with large breaks. Because the distribution of response time was unimodal (not bimodal), we could not discriminate between reporters who did and did not take breaks. Therefore, in addition to RMST, we also calculated the median time for careful interpretation (median time is not affected by very long breaks). The required time (in minutes) to complete a questionnaire is traditionally used as an indicator of the feasibility of questionnaires; however, this parameter may be less useful for eSurveys, which can be completed on personal devices. Instead, other indicators may be more useful (e.g. motion of mouse pointer).

Participants were not blinded to their assigned survey format because it was by nature visible. However, they did not see the alternate format because randomization was conducted by family. Therefore, the lack of blindness likely did not lead to any bias (e.g. the Hawthorne effect). A future study, such as a factorial randomized controlled trial, could provide further verification of our findings. More studies are needed to determine why children answered differently to adults. How children respond differently to ePROs should continue to be explored.

Our results should be interpreted keeping in mind that reporters used their own devices. The comparison in this study between narrow and wide-screened devices cannot be generalized to studies where a specific device is used by all reporters. However, this randomized study offers very practical results from realistic conditions in which the ePRO survey may be administered on different types of devices (belonging either to a clinician or reporter).

## Conclusions

Importantly, this study verified the response invariance of dynamic and basic formats of the ePedsQL; children’s response invariance to ePedsQL had not been reported prior to this study. The item non-response rates to both the basic and dynamic ePedsQL were lower than those previously reported for the paper-and-pencil version of the PedsQL. This suggests that adoption of either ePedsQL format will lower non-response rates. Using a non-response alert that does not force responders to provide an answer is an ethical way to eliminate accidental non-response. This will have the greatest impact in settings where item non-response is high. A conditional question branch is likely to be an effective way to decrease ePRO completion time only if it significantly reduces the number of questions shown to a responder. Since only 3 items were cut from the ePedsQL in the condition that a child did not attend school, the sudden appearance of the conditional question—a different type of question from the rest of the survey—may have confused responders long enough to result in a net increase in the survey completion time. We were most surprised to find that the alternate item-by-item display, which was shown to responders taking the dynamic ePedsQL on narrow screens, took more time to complete than the matrix view (which we thought would be less legible on handheld devices). More work is needed to identify device-specific effects. Overall, this randomized comparative study furthers our understanding of how humans respond to special software functions and different digital survey formats and has given us new insight on how the three tested functions might be most successfully implemented.

## Supplementary information

**Additional file 1.**

**Additional file 2.**

## Data Availability

The datasets used and/or analysed during the current study are available from the corresponding author on reasonable request.

## Abbreviations

ePedsQL: Electronic Pediatric Quality of Life Inventory
ePRO: Electronic patient-reported outcome
ΔCFI: A degree of decrease of comparative fit index
CFI: Comparative fit index
GLMM: Generalized linear mixed model
ID: Identifying digit
PedsQL: Pediatric Quality of Life Inventory
PRO: Patient-reported outcome
RMST: Restricted mean survival time
RMSEA: Root mean square error of approximation
SRMR: Standardized root mean square residual
